# Supplementary material for: Sustained and Targeted Delivery of Self-Assembled Doxorubicin Nonapeptides Using pH-Responsive Hydrogels for Osteosarcoma Chemotherapy
Source: Pharmaceutics. 2023 Feb 16;15(2):668. doi: 10.3390/pharmaceutics15020668 (PMC9961168; doi:10.3390/pharmaceutics15020668)
Supplement: Supplementary file 1 [file pharmaceutics-15-00668-s001.zip › pharmaceutics-2119611-supplementary.pdf]

Table S1. Mass spectrometry confirmation of P1 peptide.

Peptide	Molecular weight	Theoretical value	Measured value
P1	1072.26	[M+H]+ 1073.26<br>[M+2H]2+ 537.13	[M+H]+ 1073.21<br>[M+2H]2+ 537.33

Table S2. Summary of phase behavior of peptides at different pH values.

peptides	pH		
	5.8	7.4	9.2
P0	SG	UG	UG
P1	FL	SG	SG
P2	SG	UG	UG

FL: Fluid liquid; SG: Stable gel; UG: Unstable gel.

Table S3. Summary of phase behavior of peptides in different concentration.

Peptides	Concentration (mg/mL)			
	5	10	15	20
P1	VL	SG (17 min)	SG (3 min)	SG (instantly)
P2	VL	UG (3 min)	UG (3 min)	UG (3 min)

VL: Viscous liquid; SG: Stable gel; UG: Unstable gel.

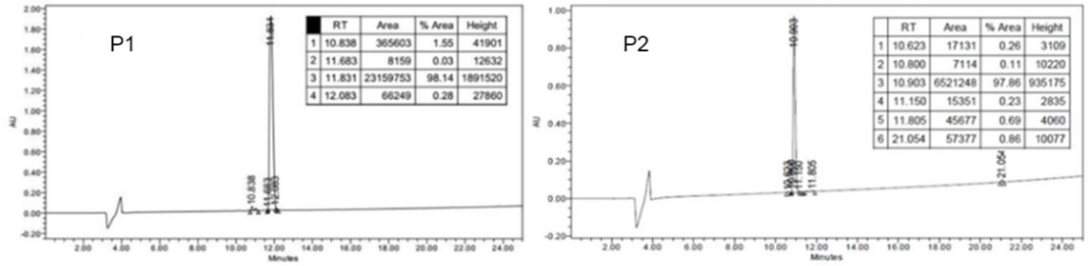

Figure S1. The UPLC chromatograms and peak data of designed nonapeptides.

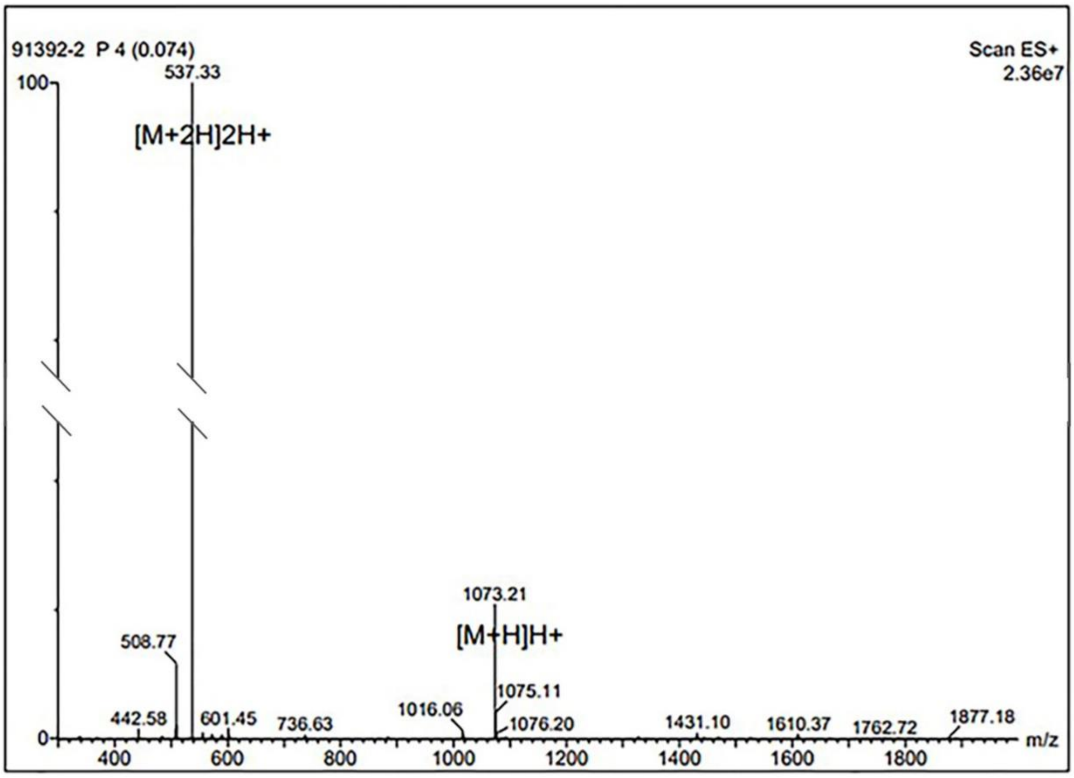

Figure S2. The mass spectrum of P1 peptide.

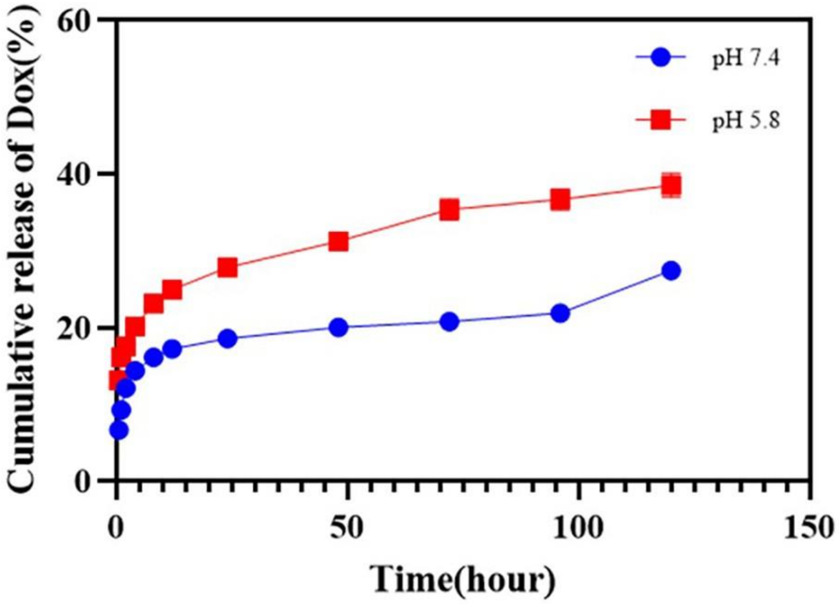

Figure S3. Cumulative release of Dox from DOX-P2 hydrogel.

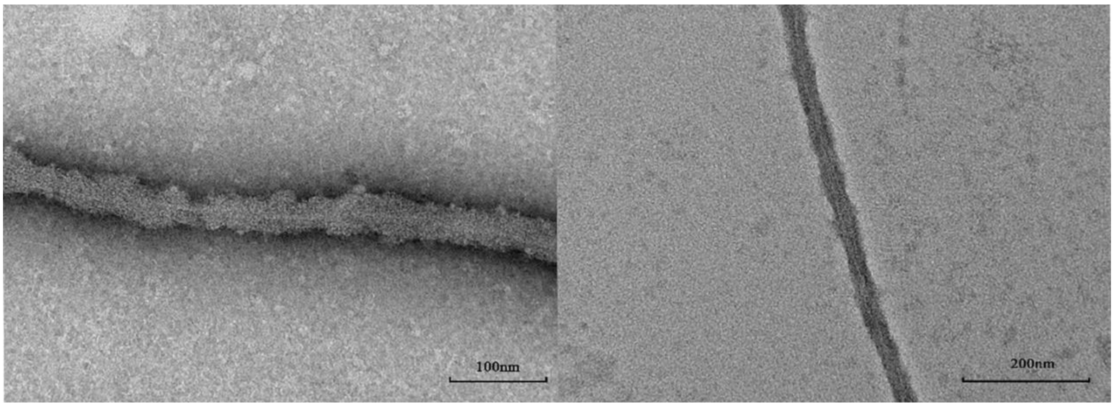

Figure S4. Transmission electron microscopy of drug loaded nanofiber.
